# Mobile Acceptance and Commitment Therapy With Distressed First-Generation College Students: Microrandomized Trial

**DOI:** 10.2196/43065

**Published:** 2023-05-15

**Authors:** Emily Brenny Kroska Thomas, Tijana Sagorac Gruichich, Jacob M Maronge, Sydney Hoel, Amanda Victory, Zachary N Stowe, Amy Cochran

**Affiliations:** 1 Department of Psychological and Brain Sciences University of Iowa Iowa City, IA United States; 2 Department of Psychiatry University of Wisconsin-Madison Madison, WI United States; 3 Department of Biostatistics The University of Texas MD Anderson Cancer Center Houston, TX United States; 4 Department of Psychiatry University of Michigan Ann Arbor, MI United States; 5 Department of Population Health Sciences University of Wisconsin-Madison Madison, WI United States; 6 Department of Mathematics University of Wisconsin-Madison Madison, WI United States

**Keywords:** acceptance and commitment therapy, randomized controlled trials, mobile health, mHealth, first-generation college students, psychological flexibility, distress, depression

## Abstract

**Background:**

Extant gaps in mental health services are intensified among first-generation college students. Improving access to empirically based interventions is critical, and mobile health (mHealth) interventions are growing in support. Acceptance and commitment therapy (ACT) is an empirically supported intervention that has been applied to college students, via mobile app, and in brief intervals.

**Objective:**

This study evaluated the safety, feasibility, and effectiveness of an ACT-based mHealth intervention using a microrandomized trial (MRT) design.

**Methods:**

Participants (N=34) were 18- to 19-year-old first-generation college students reporting distress, who participated in a 6-week intervention period of twice-daily assessments and randomization to intervention. Participants logged symptoms, moods, and behaviors on the mobile app Lorevimo. After the assessment, participants were randomized to an ACT-based intervention or no intervention. Analyses examined proximal change after randomization using a weighted and centered least squares approach. Outcomes included values-based and avoidance behavior, as well as depressive symptoms and perceived stress.

**Results:**

The findings indicated the intervention was safe and feasible. The intervention increased values-based behavior but did not decrease avoidance behavior. The intervention reduced depressive symptoms but not perceived stress.

**Conclusions:**

An MRT of an mHealth ACT-based intervention among distressed first-generation college students suggests that a larger MRT is warranted. Future investigations may tailor interventions to contexts where intervention is most impactful.

**Trial Registration:**

ClinicalTrials.gov NCT04081662; https://clinicaltrials.gov/show/NCT04081662

**International Registered Report Identifier (IRRID):**

RR2-10.2196/17086

## Introduction

### Background

Despite previous research regarding its necessity, there is a gap in access to mental health services [1-5], and the gap has been further widened by the COVID-19 pandemic [6,7]. According to Mental Health America, 24.7% of adults with mental illness report unmet needs relating to treatment [8]. Gaps in access to mental health services are further exacerbated on college campuses and among students [9,10]. The prevalence of mental health concerns and barriers to treatment on campus have been termed a crisis [11-13]. One approach to closing treatment gaps is to provide more accessible treatments via technology in order to ameliorate the current mental health crisis college students face [14-17].

One way to improve accessibility is through mobile health (mHealth) interventions. mHealth interventions have demonstrated effectiveness in a variety of conditions, including depression, anxiety, bipolar disorder, borderline personality disorder, and post-traumatic stress disorder [18-23]. Specifically, just-in-time adaptive interventions are successful in providing individuals with the support they need based on their situation and context, and technological advances provide the framework to apply these concepts to mental health [24]. Just-in-time adaptive interventions highlight the effectiveness of interventions, but also when, which, and for whom interventions are most effective, providing critical contextual sensitivity that is often missed in traditional 2-armed randomized-controlled trials. Beyond the development and evaluation of mHealth interventions, when addressing psychiatric conditions, it is important to expand beyond diagnoses and target transdiagnostic psychotherapeutic processes that extend beyond syndromes into functioning.

### Psychological Flexibility

One such process is psychological flexibility, defined as the ability to engage in behavior that is consistent with one’s values even when challenged or distressed [25,26]. Psychological flexibility is targeted in acceptance and commitment therapy (ACT), a transdiagnostic intervention with demonstrated efficacy [27].

Psychological flexibility is comprised of interrelated processes, often divided into three core pillars: (1) *openness to experiences—*willingness to make contact with emotions, thoughts, physical sensations, urges, and memories without judgment; (2) *awareness—*purposefully paying attention to the experiences of the present moment and the ability to notice the function of one’s behaviors; and (3) *engagement with values—*behavioral pursuit of personally chosen values in a consistent and flexible manner. These processes are theoretically interwoven, and attention to these processes allows for targeted intervention driven by precise case conceptualization.

Several mHealth interventions have examined the efficacy of ACT in a variety of different samples. For example, SmartQuit, a mobile app designed for smoking cessation, demonstrated higher engagement and quit rates than a non–ACT-based app [28]. In a more recent randomized controlled trial, an ACT-based mobile intervention increased the likelihood of quitting smoking [29]. Others have used both mobile apps and in-person or phone interventions [30,31]. Several studies have tested a web-based ACT intervention though not via mobile app [32-38]. Importantly, ACT has also demonstrated effectiveness in brief interventions, indicating that change in psychological flexibility can be observed even with a small “dose” [36,38-42].

### First-Generation College Students

College students are known to be at risk for mental health conditions during this developmental window, especially for depression, anxiety, and eating disorders [43]. At particular risk are first-generation college students (FGCSs), defined here as students whose parents or legal guardians have attained less education than a bachelor’s degree. Risk is demonstrated in additional stress compared to non-FGCSs, greater need for service while also seeking fewer services, and working while being a student, among other factors [44-46]. mHealth interventions are a plausible solution to address the need for both convenience and efficacy.

### Objectives of This Study

This study sought to examine the safety, feasibility, and preliminary effectiveness of an mHealth ACT intervention for FGCSs reporting distress. The goal of the intervention was to determine whether mobile ACT was safe for delivery (ie, did not worsen depression), feasibility (ie, adherence to in-app assessments), and preliminary effectiveness (the *proximal* impact of intervention). Primary outcomes included values-based and avoidance behaviors, and secondary outcomes included depressive symptoms and stress. The authors hypothesized that the intervention would be safe; participants would respond to over 60% of the in-app assessments; and the intervention would reduce avoidance behavior, depressive symptoms, and stress while increasing values-based behavior.

## Methods

### Overview

The protocol for this study and a parallel trial with patients with bipolar disorder was published [47]. To summarize, this study examined a brief 6-week mHealth intervention with FGCSs with primary effectiveness outcomes of values-based and avoidance-based behavior and secondary outcomes of depressive symptoms and stress. The microrandomized trial (MRT) included randomization to ACT intervention or no intervention twice per day after assessments were completed.

Electronic informed consent was obtained. Given the focus on depressive symptoms, suicidality was closely monitored throughout assessments. Outcomes included safety, feasibility, and effectiveness.

### Ethics Approval

The study was approved by the institutional review board at the University of Wisconsin-Madison (#2019-0819) and was registered at ClinicalTrials.gov (NCT04081662).

### Participants

FGCSs were recruited from the University of Wisconsin-Madison through advertisements and mass electronic mailings. Potential participants contacted the research team via completion of the screening survey or by phone, wherein a screening phone call was conducted. Inclusion criteria were being an adult, a full-time first- or second-year student, an FGCS, fluent in English, and having access to a smartphone. First-generation status was defined as a student whose parents had not completed a 4-year college degree. FGCSs had to report distress on 4 or more of the last 7 days that interfered with functioning when asked “Over the past seven days, on how many days did you experience distress that interfered with your ability to fulfill your responsibilities in one or more domains of life (eg, school, home, social, work, and intimate relationship)?”; this criterion was used as college students may be more likely to endorse stress than anxiety or depressive symptoms [48]. Notably, *stress* and *distress* refer to different constructs, and the authors’ aim was to recruit participants who were experiencing interference with functioning secondary to distress. Potential participants had to be willing to receive a consent form for consideration via email, as Research Electronic Data Capture (REDCap, Vanderbilt University) software was used to obtain electronic informed consent. After screening for eligibility, the research team reviewed the informed consent and study procedures with the potential participant, and if interested, the document was sent via REDCap. Recruitment for the study began in the fall semester of 2019, and a second recruitment effort occurred in the fall of 2020. The research team aimed to recruit 50 participants, but due to the COVID-19 pandemic, the number of consented participants was less than the goal (N=34). Details on how a target sample size was determined are found in the published protocol [47].

### Procedures

#### Assessment

Participants completed a baseline assessment after consenting to participate. The assessment included measures of current symptoms, stress, functioning, and psychological flexibility [47]. Daily in-app assessments were completed throughout the 6-week intervention period. At the conclusion of the intervention (day 42), participants completed a web-based assessment, including current symptoms, stress, functioning, and psychological flexibility. In addition, a mobile app engagement survey was administered. Furthermore, a 3- and 6-month follow-up survey assessed symptoms, stress, functioning, and psychological flexibility. Participants were compensated for completion of the baseline and follow-up assessments and for each week of the 6-week intervention period in which they completed at least 50% of daily in-app assessments.

#### Mobile App

After consenting to participate and completing the baseline questionnaire, participants downloaded a mobile app called Lorevimo (Log, Review, and Visualize your Mood). The app was designed by the senior author, and participants could download the app without charge from the Apple or Google Play stores. Upon opening Lorevimo, the participants set typical wake and bedtimes for weekdays and weekends. These times determined the morning and evening assessment intervals, with morning occurring 2-7 hours after waking and evening 3 hours before or 2 hours after bedtime. Participants consented to receive notifications from the Lorevimo app. Notifications were sent at the start of the interval and every 2 hours following the initial notification until symptoms were logged or it was within 30 minutes of the participant’s reported bedtime. Finally, participants watched an introductory video that introduced the ACT Matrix [49], a therapeutic tool used to help participants notice and sort emotions, thoughts, and behaviors. The video was 20 minutes in length and depicted a role play of a therapist (ET) and student (SH) walking through the ACT Matrix. At the end of the video, the therapist noted that the terminology would be used each day throughout the intervention, defining *toward* behaviors as those that move a person toward personally held values (ie, values-based behaviors), and *away* behaviors as those that function to avoid unwanted internal experiences (eg, emotions and thoughts).

#### Daily Assessments

Participants responded to twice-daily prompts in Lorevimo. Prompts were time-sensitive, and morning prompts assessed symptoms since waking, and evening prompts assessed symptoms since about lunchtime. Participants completed the Patient Health Questionnaire–2 [50], a measure of the 2 key symptoms of depression identified in the Diagnostic and Statistical Manual-5 [51] to assess depressive symptoms. Items assess dysphoria and anhedonia on an ordinal scale from 0 to 3, ranging from *absent* to *severe*. In addition, participants completed the Perceived Stress Scale- 4 [52] to assess perceived stress. Items are rated on a 0-4 ordinal scale ranging from *never* to *very often*. Finally, participants completed a 4-item ACT activity scale that was developed for the study and parallel trial. Questions are stated with the intended construct following:

1. “In a few words, what behavior are you engaged in now?” (behavioral form)
2. “Does this behavior move you toward who/what matters or away from internal experiences? [Reminder: if the behavior is both, choose which best fits.]” (behavioral function)
3. “Since [lunchtime or waking up], how much energy was consumed by trying to get rid of unwanted feelings, thoughts, and other internal experiences (eg, suppressing, distracting, avoiding)?” (avoidance behavior)
4. “Since [lunchtime or waking up], how much energy was consumed by pursuing your values? (eg, making choices that align with who you want to be or who/what matters)” (values-based behavior).

The behavioral form question was a free response, and the behavioral function was categorical (toward or away). The avoidance and values-based behavior questions were rated on a 0 to 6 ordinal scale, ranging from *none* to *all of my energy*.

#### Intervention

After completing the in-app assessment, participants were randomized to receive an intervention or no intervention (1:1). This repeated randomization occurred every time an assessment was completed. Throughout the 6-week intervention period, a participant who completed all assessments could receive up to 84 interventions. If the participant was randomized to no intervention, the app returned them to the Lorevimo homepage. If randomized to the intervention, the intervention was then randomly chosen from 84 possible prompts such that each intervention prompt was equally likely to be chosen, regardless of whether that intervention had been previously received.

The intervention prompts were developed by the first author with feedback from the research team. The prompts spanned the three core pillars of ACT [53]: awareness of internal experiences, openness to experiences, and engagement with values, discussed from here forward as awareness, openness, and engagement. The prompts were brief and in congruence with the microintervention design. Awareness intervention prompts focused on the promotion of mindful attention to context, internal experiences, and behaviors. Perspective taking and noticing the many parts of the human experience were emphasized. Sample awareness intervention prompts include: “Throughout your day, what can you notice with your five senses (sight, sound, taste, touch, smell)?” and “In what situations do you notice yourself acting on autopilot?” Openness prompts were intended to facilitate acceptance of internal experiences (eg, thoughts, emotions, physical sensations, memories) and detachment from thoughts. Sample openness prompts include: “When you experience a difficult emotion, what do you notice yourself doing to avoid or suppress the emotion?” and “When you tell yourself to not think or feel a certain way, when does it work, and when does it not work?” Engagement prompts centered on identification and clarification of personal values, consistency of actions with one’s values, and behavioral pursuit of one’s values. Sample engagement prompts include: “What is the smallest step you could take toward something that matters to you?” and “Over the past week, did you notice yourself doing something in service of what matters most, even when difficult thoughts or emotions were present?” Openness, awareness, and engagement intervention prompts were represented in equal proportion of the total 84 prompts (ie, 28 prompts in each domain).

### Statistical Analyses

Key outcomes included: (1) safety, operationalized as nonworsening in depressive symptoms from baseline to follow-up; (2) feasibility, operationalized as completion of in-app assessments; and (3) preliminary effectiveness, operationalized as the *proximal* impact of the intervention on values-based and avoidance behavior. We included sociodemographic characteristics, intervention characteristics, and in-app ratings prior to randomization as covariates in the analyses.

Two-tailed hypothesis tests were used for all analyses, and significance was defined as *P*<.05. To examine feasibility, a one-sample *z*-test assessed whether participants completed 50% of the assessments per day (1 of 2) for at least 60% of the intervention days (42 total). Safety was examined whether the change in depressive symptoms and stress was significantly different from 0 using a *z*-test, as well as using a sign test to determine whether equal proportions of individuals reported decreased depressive symptoms as those who reported increases in depressive symptoms. Effectiveness was examined using a weighted and centered least squares method [54,55]. When intervention assignment is randomized with a constant probability, as is done in this study, the estimation procedure is effectively equivalent to fitting a generalized estimating equation (GEE) with an independent working correlation structure. As in GEE, a working model is specified for the population-level mean, which in the case of an MRT is the mean of a given proximal outcome conditional on a participant completing the assessment, thus being available for randomization. We specified a linear working model for this conditional mean that consisted of an intercept, intervention delivery, time, intervention delivery × time interaction, and additional control variables predictive of missingness (these variables are described below). A model was built separately for each primary outcome (values-based behavior and avoidance behavior) and each secondary outcome (depressive symptoms and stress). The inferential target of estimation was the coefficient associated with intervention delivery and its interaction with time, representing the population’s average effect of delivering an intervention on a given outcome as a function of time and conditional on being available for randomization. A sandwich estimator was used to calculate robust standard errors [56]. Additional analyses were performed by repeating the exact estimation procedure except for changing the proximal outcome and adding interactions terms with intervention delivery.

For effectiveness, statistical analyses were powered to analyze the primary outcomes (values-based behavior and avoidance behavior). However, additional analyses were performed to analyze secondary and exploratory outcomes and interactions, and these analyses involve hypothesis tests. Multiple comparisons were not controlled for in these additional analyses, and as such, the reported *P* values should be considered nominal.

The percentage missing for primary outcomes was greater than 10%, so as prespecified in the protocol, additional variables that predicted missingness were added to the linear working model. Variables that predicted missingness were age, time of day, day in the study, count of prior completed in-app assessments, count of prior missing data points, and depressive symptoms reported immediately prior to randomization. Selection of these variables was guided by an increase of 2 in the quasi-information criterion (QIC) [57], which is similar to the commonly used BIC, but for GEEs. This was implemented using the *geepack* package in R (R Foundation) [56,58]. Due to the observed missingness, the accuracy of results relies on the validity of the missingness model.

## Results

### Study Flow

**Figure 1:**
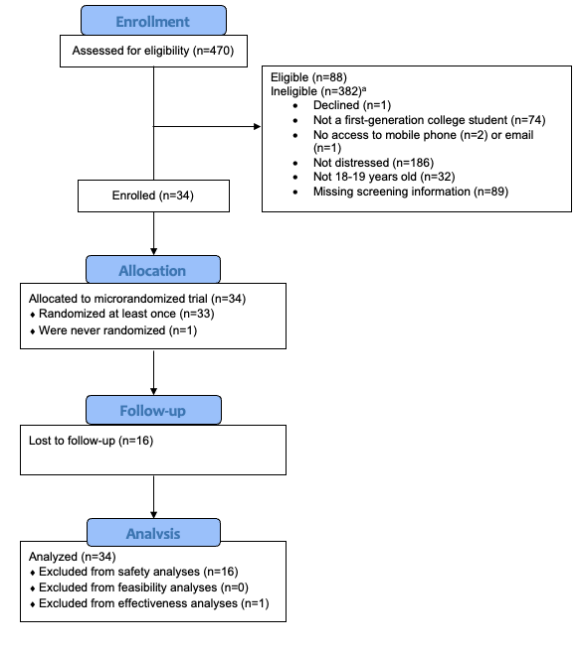
CONSORT flow diagram. ^a^Some individuals were ineligible for more than 1 reason.

A total of 470 individuals completed the web-based eligibility screening, of which 88 were eligible. Of these, 42 ultimately provided signed informed consent to participate. After consent, participants were asked to complete a baseline assessment, and 34 of the consented participants completed the baseline assessment. Following the baseline, participants were asked to download the mobile app, complete information about sleep and wake times, enable push notifications, and watch the introductory video. Of the 34 participants who completed the baseline, 33 downloaded the app and logged symptoms in the app at least once. The intervention lasted for 6 weeks, and at 3- and 6 months following the baseline, 18 participants completed the 3-month follow-up, and 15 participants completed the 6-month follow-up. For analyses, the 34 participants who completed the baseline assessment will be used to determine feasibility outcomes; the 18 participants who completed the 3-month follow-up assessments will be used to determine safety outcomes; and the 33 participants who logged symptoms at least once will be used to determine preliminary effectiveness (Figure 1).

### Sample Characteristics

Table 1 summarizes the characteristics of the sample (N=34). They had an average age of 18.53 (SD 0.53) years and were 85% (n=28) female. The majority were White (n=21, 62%) and non-Hispanic (n=31, 91%).

**Table 1:** Characteristics of the sample population (N=34).^a^

Variable		Values
Age (years), mean (SD)		18.53 (0.53)
Female, n (%)^a^		28 (85)
**Race, n (%)**		
	African American or Black	3 (9)
	Asian	7 (21)
	Caucasian	21 (62)
	More than one	2 (6)
	Other	1 (3)
	Hispanic	3 (9)
Baseline PHQ-9^b^ score, mean (SD)		10.35 (4.85)

^a^One individual reported nonbinary gender identity.

^b^PHQ-9: Patient Health Questionnaire–9.

### Safety

**Figure 2:**
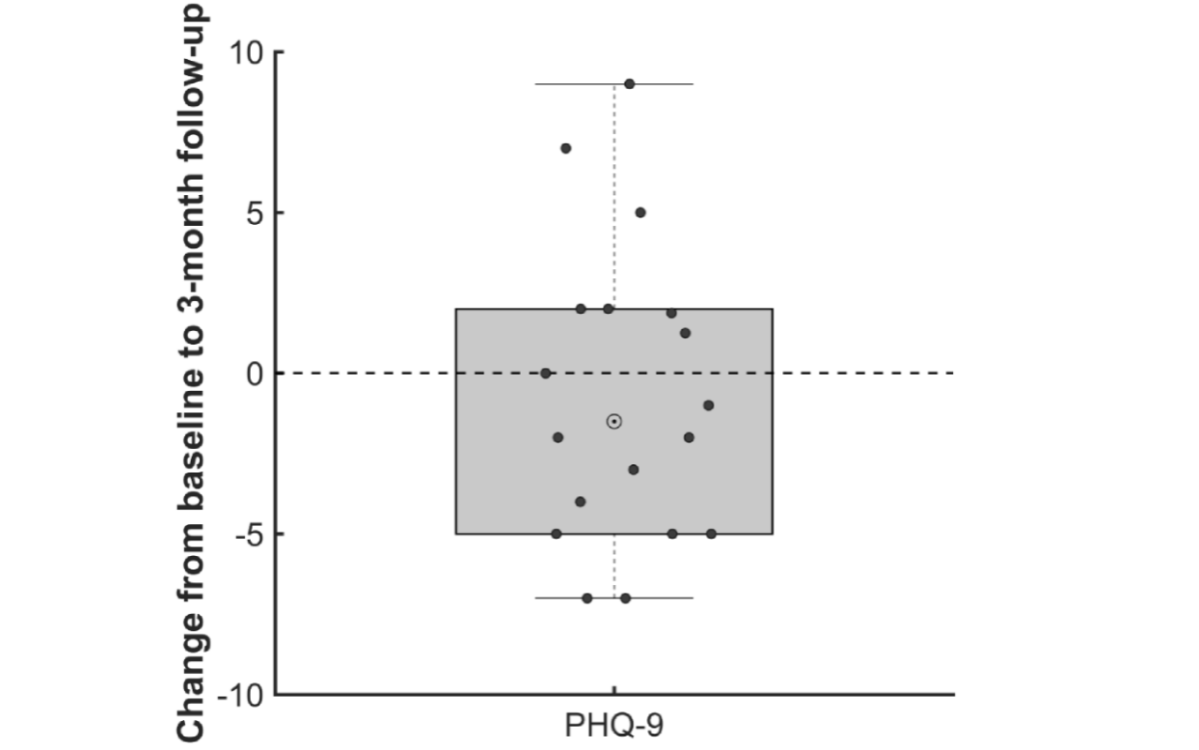
Change in Patient Health Questionnaire–9 (PHQ-9) scores from baseline to 3-month follow-up.

Analyses examined whether Patient Health Questionnaire–9 (PHQ-9) scores worsened from baseline assessment to 3-month follow-up. Figure 2 illustrates the change from baseline to 3-month follow-up in PHQ-9 scores for the 18 participants who completed the baseline and follow-up measures. Depressive symptom severity decreased slightly with an average decrease in PHQ-9 score of 0.72 points (*t*_17_=.65; *P*=.52). Using the cutoff of a score of 10 or higher on the PHQ-9 as an indication of moderate or higher depressive symptoms, 4 participants who were depressed at baseline were no longer depressed at follow-up, compared to 2 participants who were not depressed at baseline but were depressed at follow-up (67%; *z*=0.82; *P*=.41). Furthermore, 7 participants were not depressed at baseline or follow-up, leaving 5 participants who were depressed both at baseline and follow-up. Importantly, on average, it appears participants who completed the baseline and 3-month follow-up assessments did not report worsened depressive symptoms following the intervention period.

### Feasibility

Because app engagement is a primary concern with mobile interventions, our second analysis investigated whether participants were available for in-app randomization, that is, whether a participant logged their symptoms in app at 1 of the 84 time points (=2 per day×42 days). After completing consent, participants were available for randomization for an average of 57% of the time points. Excluding the 1 participant who never logged symptoms, availability increased to an average of 58% of the time points. In addition, participants were available at least once per day for an average of 71% of the days. This average was significantly larger than our prespecified target of 60% (*t*_33_=2.13; *P*=.04). Again, excluding the participant who never logged symptoms, participants were available at least once per day for an average of 73% of the days.

### Effectiveness

Models assessed whether being randomized to intervention impacted the outcome, while controlling for relevant covariates, including the number of prior assessments completed, age (18 or 19 years old), morning (0) or evening (1) assessment, and day of intervention (centered). The most proximal measurement of depression was included as a covariate to estimate mood at the time point prior to randomization. Finally, the day × intervention interaction examined whether interventions were more effective at any point in the 42-day intervention. These analyses exclude the participant who never logged symptoms, thereby making them unavailable for randomization.

Primary outcomes included energy focused on values and energy focused on avoidance. For values-focused energy, intervention predicted increased values-focused energy at the next assessment (*χ*^2^_1_=5.58; *P*=.02). Higher depressive symptoms predicted lower values-focused energy at the next assessment (*χ*^2^_1_=10.06; *P*=.002). The full model is reported in Table 2. For avoidance-focused energy, the intervention did not predict avoidance (*P*=.24). Higher depressive symptoms predicted higher avoidance-focused energy at the next assessment (*χ*^2^_1_=68.24; *P*<.001). The full model is reported in Table 3.

Secondary outcomes included depressive symptoms and perceived stress. For depression, intervention predicted lower depressive symptoms at the next assessment (*χ*^2^_1_=8.56; *P*=.003). Higher depressive symptoms predicted higher depressive symptoms at the next assessment (*χ*^2^_1_=43.97, *P*<.001). The full model is reported in Table 4. For perceived stress, intervention trended, but was not statistically significant, toward predicting decreased perceived stress (*χ*^2^_1_=3.72; *P*=.05). Moreover, higher depressive symptoms predicted higher perceived stress at the next assessment (*χ*^2^_1_=32.20; *P*<.001). The full model is reported in Table 5.

Exploratory analyses examined the specific process being targeted in the intervention (ie, engagement, awareness, and openness) as predictive of the outcomes for which the intervention had a significant effect. With values-focused energy, interventions targeting awareness (*χ*^2^_1_=11.99; *P*=.001) predicted greater values-focused energy at the next assessment. Table 2 presents the exploratory model. With depression, interventions targeting awareness (*χ*^2^_1_=9.58; *P*=.002) and openness (*χ*^2^_1_=5.08; *P*=.02) predicted lower depressive symptoms at the next assessment (Table 4). An additional exploratory model examined the interaction between intervention and depressive symptoms (at assessment just before intervention) in predicting depressive symptoms (at next assessment), finding no significant interaction (*P*=.72). Further, exploratory models investigated *for whom* the intervention may be most effective, finding no significant interaction between intervention and sex in predicting depressive symptoms and values-focused energy. Similarly, an age×intervention interaction was nonsignificant in predicting depressive symptoms and values-focused intervention. Finally, due to the small number in non-White racial groups, a categorical comparison of White and non-White participants was conducted, wherein the race×intervention interaction was investigated as a nonsignificant predictor of depressive symptoms or values-focused energy.

**Table 2:** Predictors of values-focused energy, including primary analysis and exploratory analysis of intervention type.

Predictor					Estimate			SE			*χ* ^2^_1_		*P* value			95% CI
**Primary model**																
	Evening^a^			−0.11			0.11			0.95			.33		−0.33 to 0.11	
	Prior assessment^b^			0.03			0.04			0.46			.50		−0.05 to 0.10	
	Day^c^			−0.06			0.08			0.57			.45		−0.20 to 0.09	
	Prior missing^d^			0.04			0.07			0.32			.58		−0.10 to 0.18	
	Age^e^			0.01			0.36			0.001			.98		−0.69 to 0.71	
	Depression^f^			−0.15			0.05			10.06			.002		−0.23 to −0.06	
	Intervention^g^			0.22			0.09			5.58			.02		0.04 to 0.40	
	Day×intervention			0.01			0.01			0.51			.47		−0.01 to 0.02	
**Exploratory model**																
	Evening^a^			−0.11			0.11			1.05			.31		−0.33 to 0.10	
	Prior assessment^b^			0.02			0.04			0.46			.50		−0.05 to 0.10	
	Day^c^			−0.05			0.07			0.52			.47		−0.20 to 0.09	
	Prior missing^d^			0.04			0.07			0.33			.57		−0.10 to 0.17	
	Age^e^			0.01			0.36			0.00			.98		−0.69 to 0.71	
	Depression^f^			−0.15			0.05			9.46			.002		−0.24 to −0.05	
	**Intervention^g^**															
		Engagement	0.16			0.14			1.29			.26		−0.11 to 0.42		
		Awareness	0.39			0.11			11.99			.001		0.17 to 0.62		
		Openness	0.10			0.14			0.52			.47		−0.17 to 0.38		

^a^Evening (1) indicates prior assessment was in the evening.

^b^Prior assessment is a count of assessments completed.

^c^Day was centered at 21.5.

^d^Prior missing is the count of prior missing data points.

^e^Age is coded 1 for 19 years old and 0 for 18 years old.

^f^Depression is the depressive symptoms reported at the prior assessment.

^g^Intervention (1) is randomization to intervention.

**Table 3:** Predictors of avoidance-focused energy.

Predictor	Estimate	SE	*χ* ^2^_1_	*P* value	95% CI
Evening^a^	−0.47	0.14	11.17	.001	−0.74 to −0.19
Prior assessment^b^	0.01	0.03	0.23	.63	−0.04 to 0.07
Day^c^	−0.03	0.06	0.22	.64	−0.14 to 0.09
Prior missing^d^	0.05	0.05	1.19	.28	−0.04 to 0.15
Age^e^	0.18	0.22	0.67	.41	−0.25 to 0.06
Depression^f^	0.32	0.04	68.24	<.001	0.24 to 0.40
Intervention^g^	−0.07	0.06	1.40	.24	−0.19 to 0.05
Day×intervention	−0.003	0.01	0.19	.67	−0.01 to 0.01

^a^Evening (1) indicates prior assessment was in the evening.

^b^Prior assessment is a count of assessments completed.

^c^Day was centered at 21.5.

^d^Prior missing is the count of prior missing data points.

^e^Age is coded 1 for 19-years old and 0 for 18 years old.

^f^Depression is the depressive symptoms reported at the prior assessment.

^g^Intervention (1) is randomization to intervention.

**Table 4:** Predictors of depressive symptoms, including primary analysis and exploratory analysis of intervention type.

Predictor				Estimate		SE		*χ* ^2^_1_		*P* value		95% CI
**Primary model**												
	Evening^a^		−0.34		0.16		4.33		.04		−0.66 to −0.02	
	Prior assessment^b^		0.01		0.02		0.62		.43		−0.02 to 0.05	
	Day^c^		−0.02		0.04		0.22		.64		−0.09 to 0.05	
	Prior missing^d^		0.01		0.03		0.20		.66		−0.05 to 0.07	
	Age^e^		−0.03		0.16		0.03		.87		−0.34 to 0.28	
	Depression^f^		0.47		0.07		43.97		<.001		0.33 to 0.61	
	Intervention^g^		−0.22		0.08		8.56		.003		−0.36 to −0.07	
	Day×intervention		−0.01		0.01		1.45		.23		−0.02 to 0.004	
**Exploratory model**												
	Evening^a^		−0.34		0.16		4.32		.04		−0.65 to −0.02	
	Prior assessment^b^		0.01		0.02		0.63		.43		−0.02 to 0.05	
	Day^c^		−0.02		0.03		0.35		.55		−0.09 to 0.05	
	Prior missing^d^		0.01		0.03		0.19		.67		−0.05 to 0.07	
	Age^e^		−0.02		0.16		0.02		.88		−0.33 to 0.28	
	Depression^f^		0.47		0.07		44.11		<.001		0.33 to 0.61	
	**Intervention^g^**											
		Engagement	−0.04		0.08		0.22		.64		−0.20 to 0.12	
		Awareness	−0.38		0.12		9.58		.002		−0.62 to −0.14	
		Openness	−0.23		0.10		5.08		.02		−0.43 to −0.03	

^a^Evening (1) indicates prior assessment was in the evening.

^b^Prior assessment is a count of assessments completed.

^c^Day was centered at 21.5.

^d^Prior missing is the count of prior missing data points.

^e^Age is coded 1 for 19 years old and 0 for 18 years old.

^f^Depression is the depressive symptoms reported at the prior assessment.

^g^Intervention (1) is randomization to intervention.

**Table 5:** Predictors of perceived stress symptoms.

Predictor	Estimate	SE	*χ* ^2^_1_	*P* value	95% CI
Evening^a^	−0.46	0.15	8.82	.003	−0.76 to −0.16
Prior assessment^b^	0.02	0.04	0.19	.66	−0.05 to 0.09
Day^c^	0.01	0.07	0.01	.92	−0.13 to 0.14
Prior missing^d^	−0.02	0.07	0.07	.80	−0.15 to 0.12
Age^e^	0.21	0.37	0.32	.57	−0.51 to 0.93
Depression^f^	0.65	0.12	32.20	<.001	0.43 to 0.88
Intervention^g^	−0.38	0.20	3.72	.05	−0.77 to 0.01
Day×intervention	0.01	0.01	0.18	.67	−0.02 to 0.03

^a^Evening (1) indicates prior assessment was in the evening.

^b^Prior assessment is a count of assessments completed.

^c^Day was centered at 21.5.

^d^Prior missing is the count of prior missing data points.

^e^Age is coded 1 for 19 years old and 0 for 18 years old.

^f^Depression is the depressive symptoms reported at the prior assessment.

^g^Intervention (1) is randomization to intervention.

## Discussion

### Principal Findings

Given the treatment gaps in mental health, particularly among college students in the United States, there is a substantial need for accessible, empirically based treatments. mHealth interventions are one method for translating traditional psychotherapies into a modality that may improve accessibility and reach. The present MRT examined the safety, feasibility, and preliminary effectiveness of an ACT-based mHealth intervention. Twice-daily assessments were administered in-app, and if completed, participants were randomized to intervention or no intervention. Safety analyses indicated that on average, depressive symptoms decreased from baseline to 3-month follow-up. Feasibility findings indicated that participants responded at least once per day to an in-app assessment at a greater rate (71%) than the authors’ prespecified target of 60%. The findings indicated that intervention was associated with increased values-focused energy and decreased depressive symptoms at the next assessment. Moreover, exploratory analyses indicated that awareness interventions predicted increased values-focused energy, and awareness and openness interventions predicted decreased depressive symptoms.

This study provides preliminary support for an mHealth ACT-based microintervention. The findings indicate that an intervention of this magnitude may be appropriate for FGCS reporting distress more days than not. The most important to establish in this study was that participants were, on average, responsive to in-app assessments, a prerequisite to receiving an intervention. As such, the findings herein provide strong data to support adherence to the intervention among college students. Although the intervention was deemed safe in terms of a general decrease in depressive symptoms, an intervention of this magnitude may not be sufficient. Future research should investigate *for whom* an ACT-based mHealth intervention is most helpful and perhaps examine a stepped care model, wherein the mHealth intervention is followed by an increased level of care for those who remain in need. The findings align with prior work, indicating that ACT is impactful in brief intervals [38,39,41,42], via internet-delivered interventions [32-38], via mobile app [28,29,40], and with college students [36,38,59].

Importantly, traditional randomized trials have limitations in terms of conclusions that can be drawn, wherein individuals are randomized once (eg, ACT vs treatment-as-usual). The results would, in this case, offer information about the cumulative effectiveness of the ACT sessions as compared to the treatment-as-usual condition. Microrandomized interventions, however, offer more granular and proximal information about individual interventions. Twice-daily assessments, followed by randomization, allow for examination of the impact of intervention on the assessment immediately following (eg, intervention in the morning and assessment in the evening). Beyond the effectiveness of the intervention, one can also investigate *when*, *for whom*, and *what type* of interventions are most effective. The findings indicated that interventions were not more effective as the days of intervention increased (day×intervention interaction). However, it appeared that interventions delivered after the evening assessment were more predictive of decreased avoidance-focused energy, depressive symptoms, and perceived stress, but not with increased values-focused energy. Moreover, intervention effectiveness did not vary by depressive symptoms at the assessment immediately preceding intervention (interaction), although depressive symptoms preceding intervention were significantly predictive of all outcomes in the expected direction. In terms of *for whom* the intervention is most effective, the findings indicated that the impact of intervention did not vary by age (18 and 19 years old), sex (female and male), or race (White and non-White).

Finally, exploratory analyses suggested that awareness interventions were most impactful for increasing values-focused energy, and awareness and openness interventions were most impactful for decreasing depressive symptoms. Such findings provide granular information on the tailoring of mHealth interventions that will provide a strong foundation for future studies investigating the effectiveness in a larger sample. Furthermore, increased information on for whom the intervention may be most effective will be greatly helped by diversifying the sample in terms of age, gender identity, race, and ethnicity. Moreover, future investigations may address to what extent findings are similar or different across students of different years, and the role of psychiatric history might be investigated.

It is important to note the timing of the study in relation to the COVID-19 pandemic. The first wave of participants was recruited during the fall of 2019, and as such, it is possible that some portion of the 6-week intervention, if not the 3- and 6-month follow-up assessments occurred during the rise of COVID-19 in the United States (March 2020). Another recruitment wave occurred in the fall of 2021. The COVID-19 pandemic has impacted university students' mental health [60,61]. Although the participants may have experienced increased stress over the course of the study, ACT was deemed impactful for university students during the pandemic [38]. Given the ongoing mental, physical, and social impacts of the COVID-19 pandemic, the importance of mHealth interventions for college students cannot be overstated.

### Limitations

This study has several limitations that should be considered. The study did not include a diagnostic interview to determine whether symptoms were clinically significant or warranting diagnosis, and instead, measurements relied on self-report of symptoms. Moreover, the twice-daily in-app assessments were based on short forms of validated assessments but were thus limited in scope to the specific symptoms assessed. Future research should consider assessing a wider breadth of symptoms. Assessment of values-focused energy and avoidance energy was conducted with questions designed for this study, and as such, the questions are not part of a validated scale. Future research should identify valid measures of the amount of energy one devotes to values and avoidance. In addition, twice-daily assessments may not be feasible outside of a research context, and future explorations of feasibility in real-world settings may be helpful following a larger efficacy trial. The sample was also limited to 18- and 19-year-old FGCSs reporting distress more days than not at screening, and thus the generalizability of the findings is limited. Further, many eligible students did not respond to email communications after filling the eligibility survey; future studies may consider alternative follow-up communications methods after a web-based screening survey. Finally, significant attrition occurred between the end of the intervention and follow-up assessments, and as such, safety data were based on a subset of the sample. It is unclear how these responders may differ from nonresponders in the follow-up assessment.

### Conclusions

The MRT findings presented herein provide preliminary support for the safety, feasibility, and effectiveness of a microrandomized ACT-based mHealth intervention for distressed FGCSs. Amidst large treatment gaps in the United States, particularly among college students, the identification of accessible interventions is an important step toward reducing treatment gaps and increasing the reach of empirically based interventions. Moreover, among at-risk groups, including college students generally and FGCSs specifically, interventions of this sort may be examined for preventive properties. Such an approach may improve students’ skills in navigating adversity before it arises, thereby improving resilience, awareness of one’s skills, and competencies in the management of distress.

## Appendix

Multimedia Appendix 1 CONSORT-eHEALTH checklist (V 1.6.1).

## Abbreviations

ACT: acceptance and commitment therapy
FGCS: first-generation college student
GEE: generalized estimating equation
Lorevimo: Log, Review, and Visualize your Mood
mHealth: mobile health
MRT: microrandomized trial
PHQ-9: Patient Health Questionnaire–9
